# Retrocaval Ureter as a Rare Cause of Hydroureteronephrosis: A Case Report

**DOI:** 10.7759/cureus.57042

**Published:** 2024-03-27

**Authors:** Praveen K Sharma, Arun Aram, Sakthi Ganesh Subramonian, Karpagam R K

**Affiliations:** 1 Department of Radiology, Saveetha Medical College and Hospital, Saveetha Institute of Medical and Technical Sciences (SIMATS) Saveetha University, Chennai, IND

**Keywords:** fishhook ureter, preureteral vena cava, urography, kidney, hydronephrosis, flank pain, inferior vena cava, retrocaval ureter

## Abstract

Retrocaval ureter (RCU), also known as pre-ureteral vena cava or circumcaval ureter, is a rare congenital anomaly caused by inferior vena cava (IVC) dysgenesis, leading to the right ureter coursing behind the IVC. RCU results in obstructive proximal hydroureteronephrosis, remaining asymptomatic until the third decade when hydronephrosis develops. Diagnosis relies on imaging modalities like intravenous urography (IVU), ultrasonography, computed tomography urography (CTU), magnetic resonance urography, and nuclear scintigraphy. CTU provides comprehensive 3D evaluation. We report a novel case of a 50-year-old male with RCU complicated by a concurrent distal ureteral calculus. CTU demonstrated the characteristic "S-shaped" proximal ureteral deformity and its aberrant posterior course relative to the IVC, enabling accurate preoperative diagnosis. The co-occurrence of RCU with ureteral calculus is notably rare, underlining the necessity of an exhaustive diagnostic process. The patient successfully underwent a combined surgical intervention, consisting of laparoscopic ureteroureterostomy for RCU correction and ureteroscopic lithotripsy for calculus removal, showcasing a minimally invasive approach to simultaneously address both conditions. This report underscores the significance of advanced cross-sectional imaging in diagnosing RCU and demonstrates the effectiveness of integrated minimally invasive surgical techniques in treating complex urological anomalies. By documenting this case, we contribute to the broader understanding and awareness of RCU among clinicians, potentially guiding more prompt recognition and comprehensive management of this rare condition.

## Introduction

Retrocaval ureter (RCU), also known as pre-ureteral vena cava, stands as a rare congenital anomaly where the ureter anomalously courses behind the inferior vena cava. It occurs in approximately one in 1000 individuals, predominantly in males [1,2]. RCU emerges from embryological deviations, specifically the persistence of the posterior cardinal vein [2]. This leads to external compression and obstruction of the ureter, potentially causing hydronephrosis and deterioration of kidney function if left untreated [3]. Initially, patients may remain asymptomatic, but can later experience flank pain, recurrent urinary tract infections, or progressive hydronephrosis, necessitating detailed radiological imaging for accurate diagnosis [4].

The concurrent manifestation of RCU with ureteral calculi is rare, introducing significant diagnostic and treatment challenges. This complexity underscores the need for comprehensive diagnostic evaluations and multidisciplinary surgical strategies, areas where current literature is notably sparse, especially regarding concurrent urological conditions. We report a distinctive case of a 50-year-old male diagnosed with RCU complicated by a distal ureteral calculus. Not only does this case underscore the clinical rarity and diagnostic hurdles, but it also highlights the successful implementation of a combined minimally invasive surgical strategy.

By detailing this case, we aim to highlight the crucial role of advanced cross-sectional imaging in diagnosis and demonstrate the viability of integrating minimally invasive techniques for complex urological conditions like RCU with concomitant ureteral calculus. Furthermore, by documenting and discussing this unique case, we contribute to the broader understanding and awareness of RCU among clinicians, potentially facilitating more prompt recognition and comprehensive management strategies for this rare condition.

## Case presentation

A 50-year-old male presented to the Urology Department with a three-month history of right flank pain, characterized as colicky and insidious in onset, without any episodes of hematuria. He had no history of similar episodes in the past. Despite the discomfort, his complete blood counts, blood urea, creatinine, and urine tests were all within normal ranges, indicating preserved renal function. No previous surgical history. Ultrasonography revealed mild prominence of the right pelvicalyceal system but no other significant findings. This clinical and diagnostic profile suggested an obstructive pathology, so computed tomography urography (CTU) was ordered for further evaluation.

**Figure 1:**
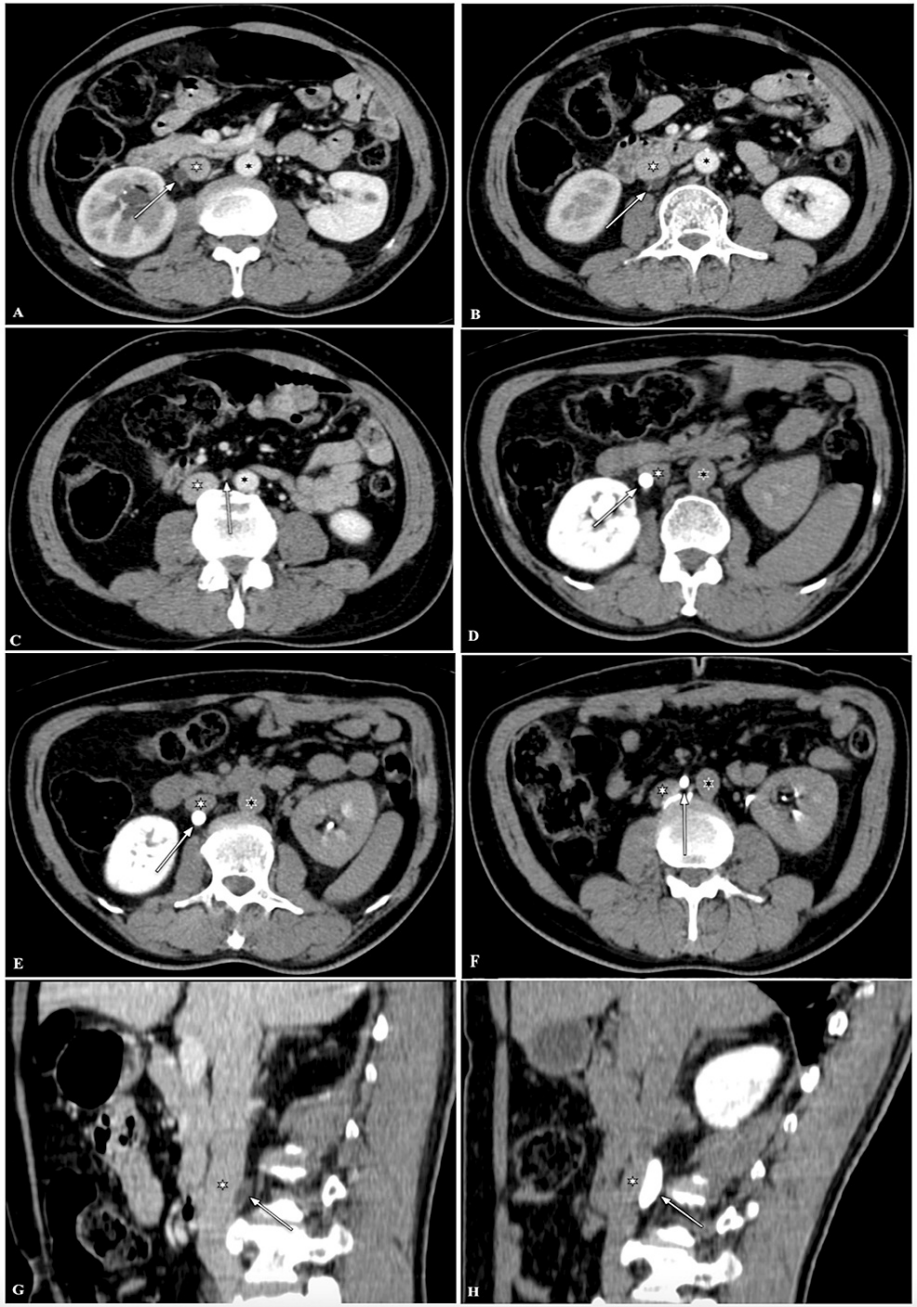
Computed tomography urography images (A-C) Axial sections in venous phase), (D-F) Axial sections in delayed phase, and (G, H) Sagittal sections in venous and delayed phases) shows the right mid ureter coursing medially (paramedian to the inferior vena cava, IVC), posterior to the IVC, and in-between the IVC and abdominal aorta (at the level of the L2-L3 vertebrae, L3 vertebra, L3-L4 vertebrae, respectively) causing mild proximal hydroureteronephrosis. Note: Right mid ureter (large white arrow), IVC (small white asterisk), and abdominal aorta (small black asterisk).

**Figure 2:**
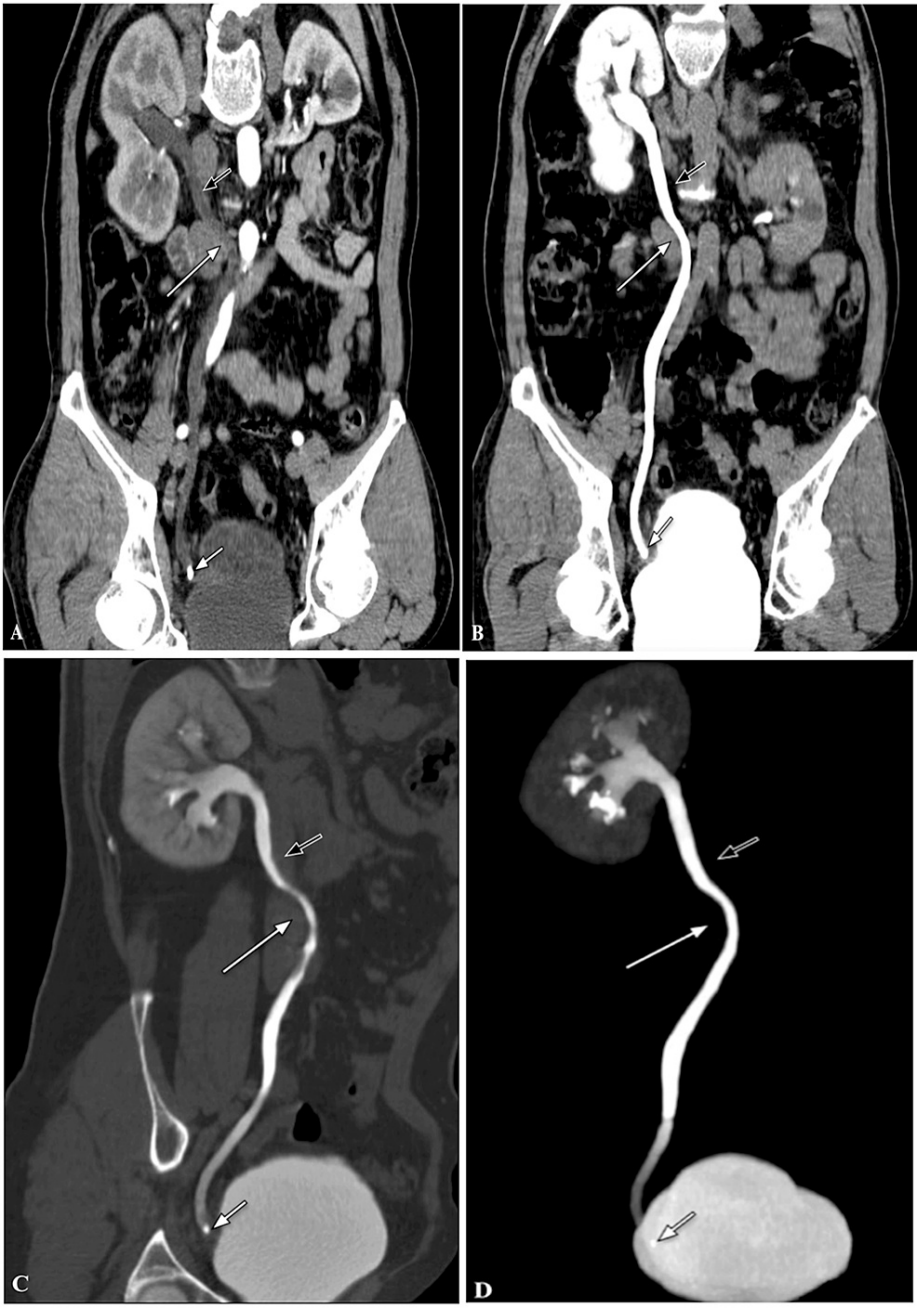
CTU with 3D curved coronal MPR (A) Venous phase, (B, C) Delayed phase, and (D) Delayed phase with Coronal Maximum Intensity Projection shows the medial deviation of the right mid-ureter with an “S”-shaped deformity of the right proximal ureter, and a radio-opaque calculus in the right distal ureter (close to the vesicoureteric junction), causing mild mid and distal hydroureter. Note: Right proximal ureter (short black arrow), right mid-ureter (long white arrow), calculus (short white arrow). CTU: Computed tomography urography, 3D: Three-dimensional, MPR: Multi-planar reconstruction.

Computed tomography urography (CTU) in venous and delayed phases showed the right mid-ureter coursing medially (para-median to IVC), posteriorly to IVC, and in-between IVC and Abdominal Aorta (at the level of L2-L3 vertebrae, L3 vertebrae, and L3-L4 vertebrae, respectively), causing mild proximal hydroureteronephrosis (due to kinking of the ureter by extrinsic compression against IVC) (Figures 1A-1H).

(CTU) with three-dimensional (3D) Curved Multi-Planar Reconstruction (MPR) in the venous and delayed phases, and Maximum Intensity Projection (MIP) in the delayed phase, showed a medial deviation of the right mid-ureter (at the level of the L3 vertebra) with an "S"-shaped deformity of the proximal ureter (due to kinking of the ureter by extrinsic compression against the IVC) and a focal radio-opaque obstructive calculus in the right distal ureter (~ 12 mm from the vesicoureteric junction), measuring 5 x 4 x 7 mm (anterior-posterior (AP) x transverse (TR) x cranio-caudal (CC)), causing upstream mild mid and distal hydroureter (Figures 2A-2D).

**Figure 3:**
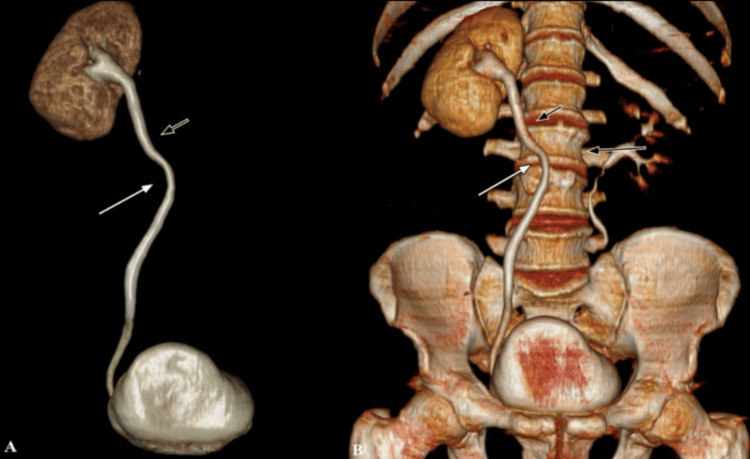
CTU with 3D coronal VR Coronal VR in the delayed phase shows the medial deviation of the right mid ureter (at the level of L3 vertebra) with an “S” shaped deformity of the right proximal ureter. Note: Right proximal ureter (short black arrow), Right mid ureter (long white arrow), L3 vertebra (long black arrow). CTU: Computed tomography urography, 3D: Three-dimensional, VR: Volume rendered.

CTU with 3D volume rendering (VR) in the delayed phase showed a medial deviation of the right mid-ureter with an "S"-shaped deformity of the proximal ureter (Figure 3). A diagnosis of symptomatic retrocaval ureter (or pre-ureteral vena cava) was made.

Given the diagnosis of symptomatic retrocaval ureter and ureteral calculus, a laparoscopic ureteroureterostomy was performed to correct the anatomical anomaly, concurrently with ureteroscopic lithotripsy to remove the calculus. Post-operatively, the patient was closely monitored for any complications and received pain management. The follow-up plan included stent removal and imaging assessments to ensure successful recovery and prevent recurrence. Lifestyle modifications and increased hydration were advised to avert future stone formation, with annual follow-ups scheduled to monitor renal function and symptoms.

## Discussion

Retrocaval ureter, also known as pre-ureteral vena cava, is a rare congenital anomaly with an incidence reported to be approximately one in 1000 people [5], with a male predominance and a male-to-female ratio of 2.8:1 [6]. It has an incidence of 0.06-0.17% worldwide. When accompanied by partial or complete situs inversus or IVC anomalies such as left IVC or double IVC, the retrocaval ureter is usually observed on the right side and rarely on the left [6,7]. Individuals with this congenital anomaly remain asymptomatic until their third and fourth decades of life when they develop symptoms due to hydronephrosis.

During fetal development between weeks four and eight, the posterior cardinal, subcardinal, and supracardinal veins give rise to the inferior vena cava (IVC). The right sub-cardinal vein typically forms the pre-renal portion of the IVC, with an anastomosis between the right sub-cardinal and supracardinal veins forming the renal section of the IVC [2]. However, in cases of retrocaval ureter, an anomalous formation of the right posterior cardinal vein occurs instead of the right sub-cardinal vein, resulting in the development of the renal section of the IVC. This leads to the ureter becoming posteriorly trapped when originating from the ventrally positioned sub-cardinal vein, forming the pre-ureteral vena cava [8]. Due to the ureter's kinking, a ureteric segment adynamic or compression against the IVC or psoas muscle leads to proximal hydroureteronephrosis. Thus, the right ureter courses posterior to the IVC, then between the IVC and abdominal aorta, continuing to course downward anterior and lateral to the IVC. Earlier, it was considered a ureteric development aberration; however, recent studies in embryology have led to its being viewed as an inferior vena cava aberration [8].

Retrocaval ureter, also known as pre-ureteral vena cava, presents in two clinical types: low loop (Type 1) and high loop (Type 2), each with distinct imaging characteristics and clinical presentations. Type 1, the more common variant, accounts for approximately 90% of cases [9]. It is characterized by a distinctive "fishhook" or S-shaped appearance of the ureter on imaging. This shape persists until the point of blockage, typically located at a distance from the side edge of the abnormally positioned inferior vena cava (IVC), at the level of the L3 vertebra. At this juncture, the ureter exhibits a medial bend and curves upwards towards the pedicle of the vertebral body, mimicking a "fish hook." About 50% of patients with Type 1 retrocaval ureter show significant medial displacement of the middle ureteric segment and moderate to severe proximal hydroureteronephrosis, often accompanied by varying degrees of right hydronephrosis due to compression from the aberrant vessel [9,10].

**Figure 4:**
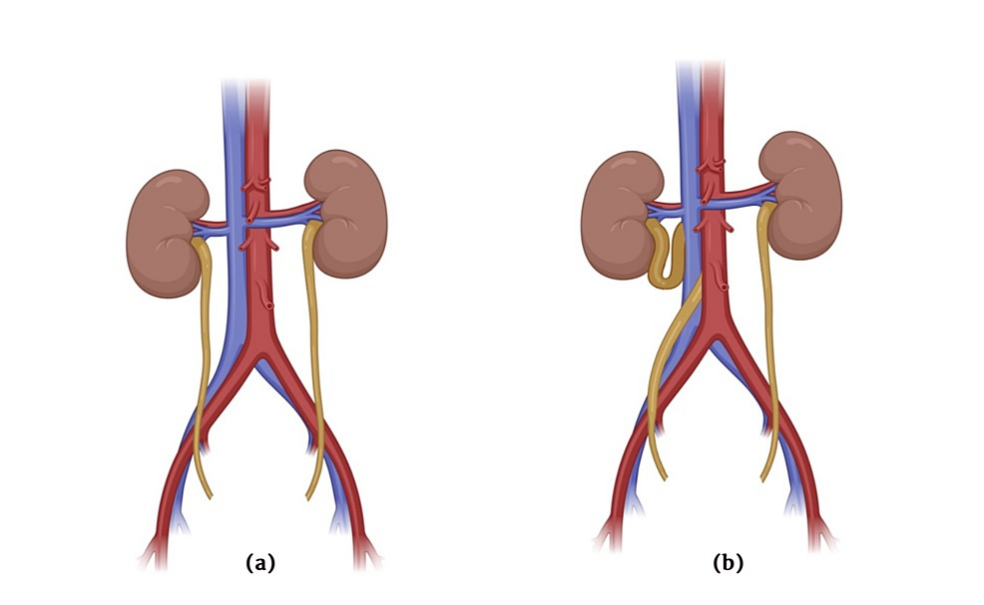
Pictorial representation (a) Shows right ureter coursing normally and (b) Shows right ureter coursing posterior to inferior vena cava (IVC). Images created by the authors.

Type 2, or the high loop variant, is rare, making up around 10% of cases [8,10]. It features a smooth, sickle-shaped curve of the right proximal ureter, with the obstruction site located at the outer edge of the L3 vertebra. This results in little or no concurrent upper urinary tract dilation, differing significantly from the severe medial displacement or significant hydroureteronephrosis commonly observed in Type 1 [10]. Patients typically diagnosed with a retrocaval ureter are in their second to fourth decades of life [11]. Moreover, the occurrence of calculi may sometimes coexist with this condition, further complicating its management and treatment [12]. The appropriate term for accurately describing the anomaly is pre-ureteral vena cava. The ureter takes a medial deviation, passing behind the inferior vena cava (IVC), and then curves around and crosses in front of it, changing its path from a medial to a lateral direction before continuing distally towards the urine bladder. The renal pelvis and proximal ureter appear dilated and elongated in an S-shaped or fishhook appearance before passing behind the IVC (Figures 4a, 4b).

Flank discomfort is the most frequently observed symptom of pre-ureteral vena cava [13,14]. This pain, which can be sporadic, dull, and throbbing, is typically caused by a blockage in the ureter and the resulting fluid buildup in the kidney (hydronephrosis). Other clinical manifestations include recurrent urinary tract infections and hemorrhage. The presence of kidney stones and a severe infection in the kidney may further worsen the situation.

The presence of an abnormal blood vessel can lead to compression of the right ureter, causing varying degrees of hydronephrosis and non-specific clinical symptoms. Imaging holds the key to diagnosis. The radiological diagnosis of retrocaval ureter has evolved from intravenous urography (IVU), venacavography, and ureteral catheterization. The presence of the retrocaval ureter in IVU is characteristic and strongly suggestive of the diagnosis, albeit not definitive [15]. Computed tomography urography (CTU) has become the preferred imaging technique for assessing the source of hydronephrosis, especially when the location and origin of obstruction are uncertain after the initial intravenous urography (IVU) study [16].

Computed tomography urography (CTU) offers a comprehensive evaluation, revealing the ureter's abnormal dorsal position, pinpointing the precise level at which it deviates medially, delineating the extent of compression exerted by the aberrant vessel on the right ureter, depicting the ureter's course behind the inferior vena cava (IVC), assessing the degree of pelvicalyceal system dilatation proximal to the obstruction, providing an indication of the kidney's excretory capability, and serving as a valuable roadmap to guide surgical intervention, should it be warranted. CTU surpasses alternative methods, such as magnetic resonance urography (MRU), due to its convenient accessibility, affordable price, exceptional sensitivity, specificity, and time-efficient examination duration [17].

Retrocaval ureter is a rare congenital anomaly where imaging studies are usually sufficient for an accurate pre-operative diagnosis, which is essential for surgical intervention or correction based on the clinical presentation, severity of hydronephrosis, and renal function impairment. The surgical management of the retrocaval ureter includes both open and laparoscopic surgical techniques. The procedure involves reconnecting the ureters using a double-J stent, with the option of removing the narrowed retrocaval segment. It also includes dividing the enlarged renal pelvis and reattaching it, as well as either tying or cutting the inferior vena cava, with the possibility of reconnecting it, or nephrectomy in the presence of a non-functional kidney, trans-peritoneal or retroperitoneal laparoscopic ureterolysis, and reconstruction of the retrocaval ureter [18,19].

The differential diagnosis for the observed symptoms and ultrasound findings can be categorized based on the location of the ureteral involvement. For pathologies involving the upper ureter, considerations include retroperitoneal fibrosis and various retroperitoneal masses. Retroperitoneal fibrosis is a rare disorder characterized by the development of fibrous tissue in the retroperitoneum, which can encase and obstruct the ureters. Retroperitoneal masses, which could range from benign to malignant neoplasms, may similarly exert pressure on the ureters, leading to obstruction and hydronephrosis. In the case of lower ureter involvement, differential diagnoses encompass lymphadenopathy, which involves the enlargement of lymph nodes that could compress the ureter; bladder diverticulum, an outpouching of the bladder wall that may impact ureteral function; complications post-surgical interventions in the pelvic area that might obstruct the ureter; and pelvic lipomatosis, a rare condition characterized by an overgrowth of fatty tissue in the pelvis that can displace or compress the urinary structures. Each of these conditions can present with symptoms similar to those seen in our patient, including flank pain and signs of urinary obstruction without necessarily leading to hematuria.

## Conclusions

Retrocaval ureter is a rare congenital anomaly that can lead to urinary obstruction and impaired renal function if left untreated. This case highlights the importance of advanced cross-sectional imaging like computed tomography urography in accurately diagnosing and characterizing retrocaval ureter, particularly when concurrent urological conditions like ureteral calculi are present. The successful integration of minimally invasive surgical techniques, including laparoscopic ureteroureterostomy and ureteroscopic lithotripsy, allowed for comprehensive management of both the anatomical abnormality and obstructing calculus in this patient. By reporting this unique case, we aim to raise awareness among clinicians about this rare condition and emphasize the potential for combining minimally invasive approaches in complex urological cases. Prompt recognition through appropriate imaging and a multidisciplinary treatment approach is crucial for preserving renal function and avoiding long-term complications in patients with retrocaval ureter, especially when accompanied by additional urological pathologies. This case underscores the importance of maintaining a high index of suspicion, utilizing advanced imaging modalities, and considering minimally invasive surgical strategies in the management of retrocaval ureter and its associated complications.
